# Comparison of disinfection effect between benzalkonium chloride and povidone iodine in nasotracheal intubation: a randomized trial

**DOI:** 10.1186/s12871-019-0839-y

**Published:** 2019-08-31

**Authors:** Aiji Sato-Boku, Keiji Nagano, Yoshiaki Hasegawa, Yuji Kamimura, Yoshiki Sento, MinHye So, Eisuke Kako, Masahiro Okuda, Naoko Tachi, Hidekazu Ito, Yushi Adachi, Kazuya Sobue

**Affiliations:** 1 Department of Anesthesiology, Aichi Gakuin University School of Dentistry, 2-11 Suemori-dori, Chikusa-ku, Nagoya, 464-8651 Japan; 2 Department of Oral microbiology, School of Dentistry Health Sciences University of Hokkaido 757 Kanazawa, Ishikari-Tobetsu, Hokkaido 061-0293 Japan; 3 Department of Microbiology, Aichi Gakuin University School of Dentistry, 1-100 Kusumotocho, Chikusa-ku, Nagoya, 464-8650 Japan; 4 Department of Anesthesiology and Intensive Care Medicine, Nagoya City University Graduate School of Medical Sciences, 1 Kawasumi, Mizuho-cho, Mizuho-ku, Nagoya, 467-8601 Japan; 5 Department of Anesthesiology, Aichi Developmental Disability Center Central Hospital, 713-8 Kagiya-cho, Kasugai-city, Aichi 480-0392 Japan; 6 Department of Anesthesiology, Nagoya University Graduate School of Medicine, 65 Tsurumaicho, Showaku, Nagoya, 466-8550 Japan

**Keywords:** Nasotracheal intubation, Benzalkonium chloride, Povidone iodine, Bacteremia

## Abstract

**Background:**

Nasotracheal intubation can potentially result in microbial contamination from the upper respiratory tract to the lower respiratory tracts. However, an ideal nasotracheal disinfection method is yet to be determined. Therefore, we compared the disinfection effects between benzalkonium chloride and povidone iodine in nasotracheal intubation.

**Methods:**

Overall, this study enrolled 53 patients aged 20–70 years who were classified into classes 1 and 2 as per American Society of Anesthesiologists-physical status and were scheduled to undergo general anesthesia with NTI. Patients who did not give consent (*n* = 2) and who has an allergy for BZK or PVI were excluded from the study. The patients were randomly divided into two groups on the basis of the disinfection method: BZK (*n* = 26, one patient was discontinued intervention) and PVI (*n* = 25). 50 patients were assessed finally.

The subjects’ nasal cavities were swabbed both before (A) and after disinfection (B), and the internal surface of the endotracheal tube was swabbed after extubation (C). The swabs were cultured on Brain heart infusion agar and Mannitol salt agar. The number of bacteria per swab was determined and the rates of change in bacterial count (B/A, C/B) were calculated. The growth inhibitory activity of the disinfectants on *Staphylococcus aureus* were also investigated in vitro.

**Results:**

Although the initial disinfection effects (B/A) were inferior for benzalkonium chloride compared with those for povidone iodine, the effects were sustained for benzalkonium chloride (C/B). In the in vitro growth inhibitory assay against *S. aureus*, benzalkonium chloride showed higher inhibitory activity than povidone iodine.

**Conclusion:**

Although both disinfectants were inactivated or diffused/diluted over time, benzalkonium chloride maintained the threshold concentration and displayed antimicrobial effects longer than povidone iodine; therefore, benzalkonium chloride appeared to show a better sustained effect. Benzalkonium chloride can be used for creating a hygienic nasotracheal intubation environment with sustained sterilizing effects.

**Trial registration:**

UMIN-CTR (Registration No. UMIN000029645). Registered 21 Oct 2017.

## Background

Nasotracheal intubation (hereafter referred to as “NTI”) is frequently necessary during dental, maxillofacial, and oropharyngeal surgeries. This method is also useful while operating on patients with respiratory insufficiency, patients who require long-term maintenance of the airway in the intensive care unit and patients in whom orotracheal intubation is difficult because of trismus. However, some complications associated with NTI include epistaxis [1, 2], bacteremia [3], retropharyngeal perforation [4], and partial or complete obstruction of the tube [5, 6]. NTI complications are some of the many causes of anesthesia-related mortality [7].

Although several effective preventive measures against epistaxis and retropharyngeal perforation have been reported [8–10], an effective disinfection method during NTI is yet to be determined. In fact, dental procedures under general anesthesia with NTI demonstrate a higher incidence of bacteremia compared with those conducted under local anesthesia [3]; moreover, patients with prosthetic heart valves, immunodeficient patients, diabetic patients, and patients taking steroids are at an increased risk of bacteremia, and such patients require antibiotic prophylaxis [11]. Reports describing the presence of NTI-related bacteremia also exist [11, 12]. Bacteremia is likely caused by transferring intranasal bacteria into the respiratory tract. Therefore, the disinfection of the nasal mucosa before nasal intubation is crucial for avoiding the contamination of respiratory organs by nasal microorganisms.

In Japan, benzalkonium chloride (hereafter referred to as “BZK”) and povidone iodine (hereafter referred to as “PVI”) are generally used as NTI disinfectants. A comprehensive literature search was performed using PubMed, the Cochrane Central Register of Controlled Trials and EMBASE. However, to the best of our knowledge, the authors were unable to identify any previous reports comparing the outcomes of disinfection effects between these two disinfectants. Therefore, we investigated the disinfection effects of BZK and PVI when used for disinfection in NTI.

## Methods

### Ethics approval and consent to participate

This study was approved by the Ethics Committee at the School of Dentistry, Aichi Gakuin University (Approval No. 495) and was registered prospectively in the UMIN-CTR as a clinical trial on 21 Oct 2017. (Registration No. UMIN000029645). Our study adhered to CONSORT guidelines. The first patient was recruited and registered on 23 Oct 2017 (https://upload.umin.ac.jp/cgi-bin/ctr/ctr_view_reg.cgi?recptno=R000033873). We obtained written informed consent from all patients after providing them with adequate explanation regarding the research aims.

### Subjects

**Fig. 1:**
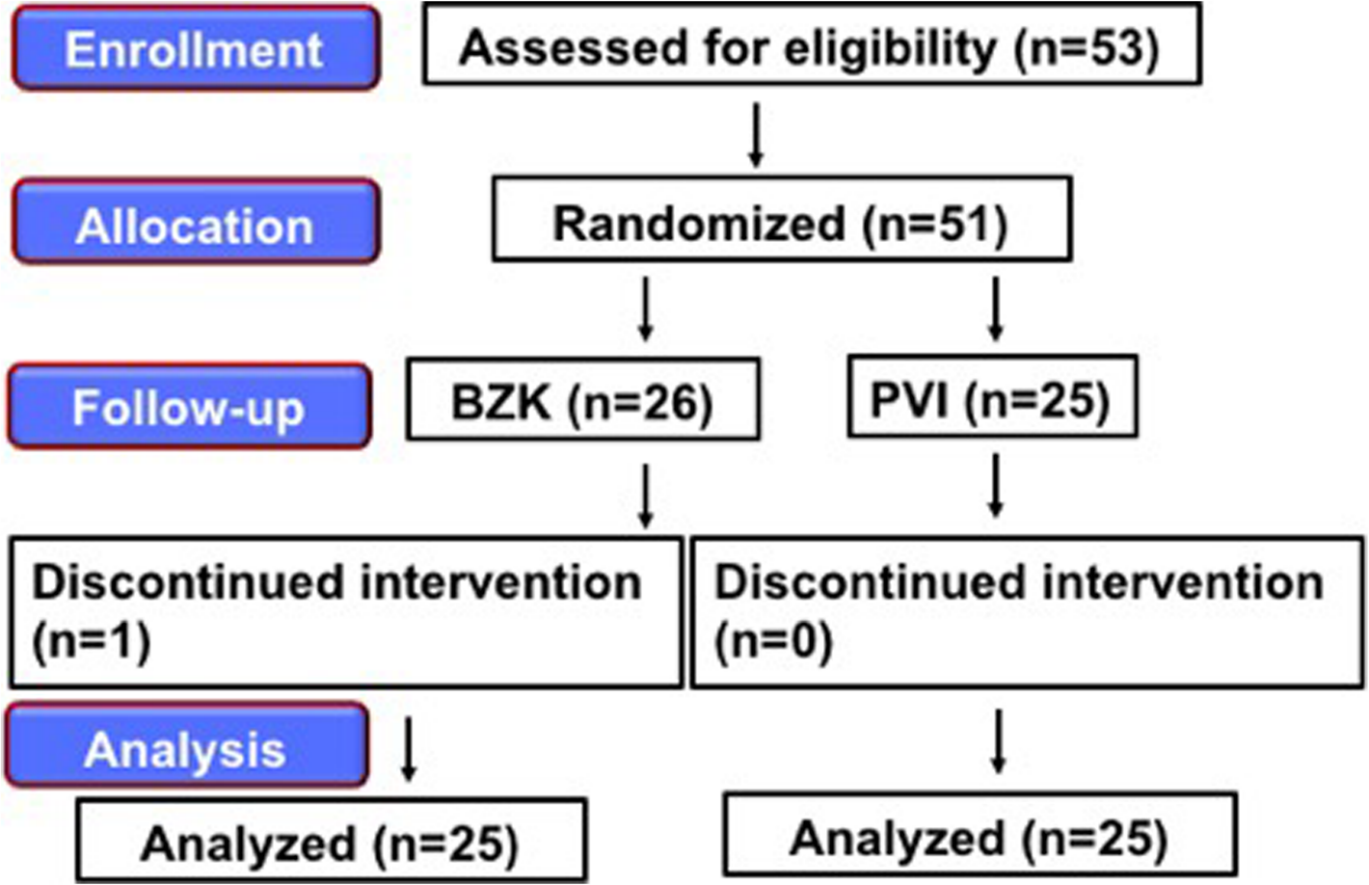
Consolidated Standards of Reporting Trials (CONSORT) recommended description of patient recruitment

Overall, this study enrolled 53 patients aged 20–70 years who were classified into classes 1 and 2 as per American Society of Anesthesiologists-physical status (hereafter referred to as “ASA-PS”) and were scheduled to undergo general anesthesia with NTI. Patients who did not give consent (*n* = 2) and who has an allergy for BZK or PVI were excluded from the study. The patients were randomly divided into two groups on the basis of the disinfection method: BZK (*n* = 26, one patient was discontinued intervention) and PVI (*n* = 25). 50 patients were assessed finally (Fig. 1).

### Anesthesia, sample collection, and microbial count

The same method of anesthesia was employed for all patients. No premedication was administered. After a patient walked independently to the operating theater, the standard vital monitors (electrocardiogram, blood pressure and oxygen saturation) were monitored. Anesthesia was induced using propofol (1–2 mg/kg), remifentanil (0.2 μg/kg/min.), and fentanyl (100 μg), with rocuronium (0.6 mg/kg) used as a neuromuscular blocking agent. Until the effects of the neuromuscular blocking agent became apparent, mask ventilation was implemented for all patients using 100% oxygen. While mask ventilation was being performed, the subjects’ inferior nasal passage was swabbed with a sterile cotton swab before disinfection (A). The subjects’ nasal cavities and inferior nasal passages were adequately disinfected twice each using a sterile cotton swab with BZK (ZALKONIN® SOLUTION 0.025, Kenei Phamaceutical Co., Ltd., Osaka) or PVI (POVIDONE-IDOINE SOLUTION 10% ⌈MEIJI⌋ Nitto Medic Co., Ltd., Toyama). BZK and PVI were applied at normal clinical concentrations of 0.025 and 5%, respectively. Next, the patients’ inferior nasal passages were swabbed again with sterile cotton swabs after disinfection (B). Then, tramazoline nasal drops were administered to the nasal cavity and NTI was conducted after the muscle relaxant was observed to take effect. In all cases, we maintained general anesthesia using Total Intra Venous Anesthesia. After the surgery, the internal surface of the endotracheal tube (inner surface 1 cm from the tip of the tube) was swabbed immediately after extubation (C). We focused on how much bacteria that invaded from the upper airway to the lower airway during intubation was suppressed by long-term disinfection effect. If we swabbed the outer surface of the endotracheal tube, we could not avoid contamination by nasal bacteria during extubation. Therefore, we swabbed the the internal surface of the endotracheal tube.

After collecting the specimens, only the swab head was cut off. The samples from (A) and (C) were placed in 10 ml of sterile physiological saline and the samples from (B) were placed in 40 ml of sterile physiological saline to dilute the disinfectants (BZK or PVI) that were absorbed by the swab. The samples were refrigerated and submitted for examination within six hours as described in the following sections.

Viable microbes in the swab samples were measured using a culture method. The samples were vigorously vortexed at maximum speed for 30 s to extract the microbes from the swab head in saline. After the swab heads were removed, the samples were centrifuged at 4000×*g* for 15 min at 4 °C to concentrate the extracted microbes, and then the precipitates were suspended in 1 ml of saline. The precipitates were serially diluted, and 50 μl of the dilutions was spread on agar plate medium. Brain heart infusion agar (hereafter referred to as “BHI”, Becton, Dickinson and Company, Franklin Lakes, NJ, USA) was used for assessing the total number of microbes. Mannitol Salt Agar (hereafter referred to as “MTS”, Nissui Pharmaceutical Co., Ltd., Tokyo, Japan) was used for detecting *Staphylococcus*. After culturing at 37 °C for 24 h under aerobic conditions, the colonies were counted and were expressed as colony-forming units (hereafter referred to as “CFU”).

### Assay for minimum inhibitory concentration (hereafter referred to as “MIC”)

The MICs of the gram-positive bacterium *S. aureus* strain FDA 209P JC-1 against BZK and PVI were examined using both BHI agar and broth media. Approximately 10^6^ CFU of bacterial cells were inoculated in the media and cultivated at 37 °C for 24 h under aerobic conditions. The MICs were visually determined.

### The evaluation of parameters

The preoperative patient attributes of sex, age, anesthesia time, and patient distribution after extubation were evaluated. The numbers of bacteria (in CFU) in the samples from (A), (B), and (C) per cotton swab were assessed, and the rates of change in bacterial count (B/A, C/B) were calculated. The MICs were also visually determined.

### Statistical analysis

We calculated the required minimum number of samples (*n* = 46 cases; BZK group, 23 cases; PVI group, 23 cases; effect size, 0.47; α-error, 0.05; power, 0.95). The effect size was calculated on the basis of the statistical results of a pilot study in which the patient distribution for the change in the number of bacteria after disinfection was used as a standard [BZK group, 10 cases; PVI group, 10 cases]. As application of statistical tests in the absence of reliable sample size calculation decrease its weightage, we calculated our final sample size as follows. The dropout rate in a preliminary study was 0.05. If an R dropout rate is expected, a simple but adequate adjustment is provided by N_d_ = N/(1-R)^2^ where N is sample size calculated assuming no dropout and N_d_ that required with dropouts [13]. Therefor our adjustment was 50.9 and 50 patients were assessed finally. Student’s t test was used for assessing the effects of age and anesthesia time. Chi-square independence test m × n contingency table was used for assessing sex and patient distributions. Based on the results of QQ plot from the sample (A), (B) and (C), Mann–Whitney U test was used for the number of bacteria found in the samples from (A), (B), (C) and the rates of change in bacterial count (B/A, C/B). The level of statistical significance was set at *p* < 0.05.

## Results

From October 2017 to December 2017, 53 patients were selected as subjects for this study. Figure 1 shows the consort flow diagram. Fifty-one subjects were randomly assigned into two groups on the basis of the disinfection method used: BZK and PVI. One subject dropped out during the trial.

Table 1 shows patient sex, age, and anesthesia time. No statistical differences in any parameters were observed between the two groups.

**Table 1:**
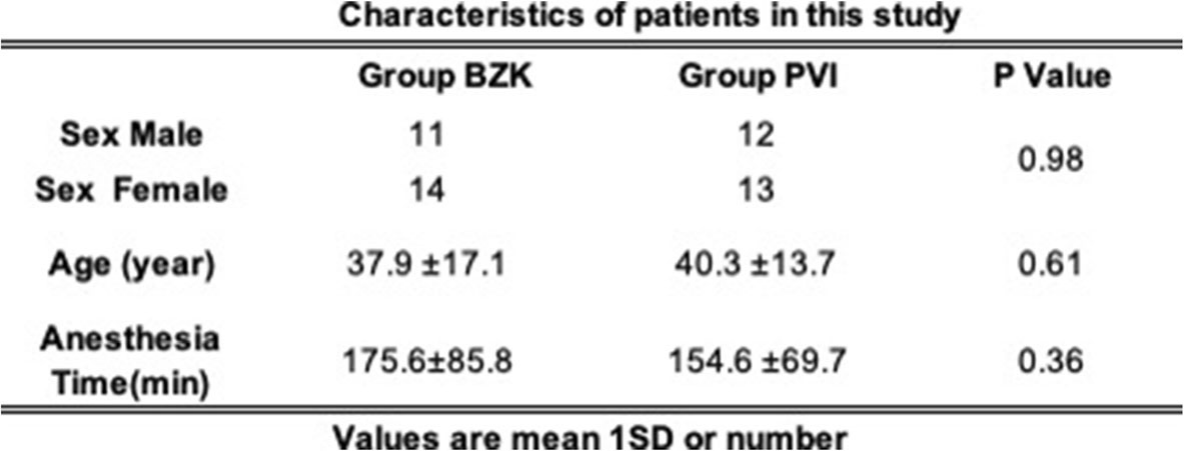
Characteristics of patients in this study

For examining the total number of bacteria, we used BHI for the general bacterial culture (Table 2). The number of nasal bacteria before disinfection (A) was equivalent in the BZK and PVI groups (30,000 and 50,000 CFU/swab, respectively) and no statistically significant differences were noted. However, individual patient differences were large and ranged from 1900 to 400,000 CFU/swab. After disinfection (B), the bacterial numbers of 1300 CFU/swab for BZK and 20 CFU/swab for PVI were reported, which correspond to a difference of 65 times (*p* = 0.00005). The rate of change (B/A) was also significantly lower for PVI than for BZK. Conversely, postoperatively (C), both groups reported a median of 1900 CFU/swab. Compared with the samples from (B), there was barely any change in the number of bacteria in the samples from (C) after BZK treatment, whereas after PVI treatment, an approximately 100-fold proliferation was observed: The rate change (C/B) for BZK and PVI was100 and 9867, respectively (*p* = 0.002).

**Table 2:**
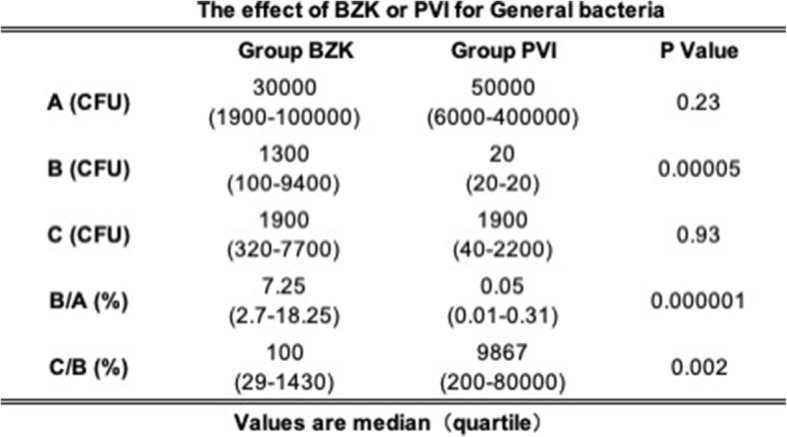
The effect of BZK or PVI for General bacteria

Table 3 shows the number of bacteria in the samples from (A), (B), and (C) in MTS and the rates of change for B/A and C/B. The number of nasal bacteria before disinfection (A) was equivalent in patients in the BZK and PVI groups (10,000 and 7000 CFU/swab, respectively) and there was no statistically significant difference noted. However, the individual differences were large and ranged from 900 to 30,000 CFU/swab. After disinfection (B), bacterial numbers of 200 CFU/swab for BZK and 20 CFU/swab for PVI were reported, and a difference of 100 times was confirmed (*p* = 0.002). The rate of change (B/A) was significantly lower after PVI disinfection than after BZK disinfection. Conversely, postoperatively (C), both groups reported an identical median of 20 CFU/swab. Compared with the samples from (B), the number of bacteria found in the samples from (C) decreased after BZK disinfection, whereas an increase in the number of bacteria in the samples from (C) after PVI disinfection was observed, when compared between their quartiles.

**Table 3:**
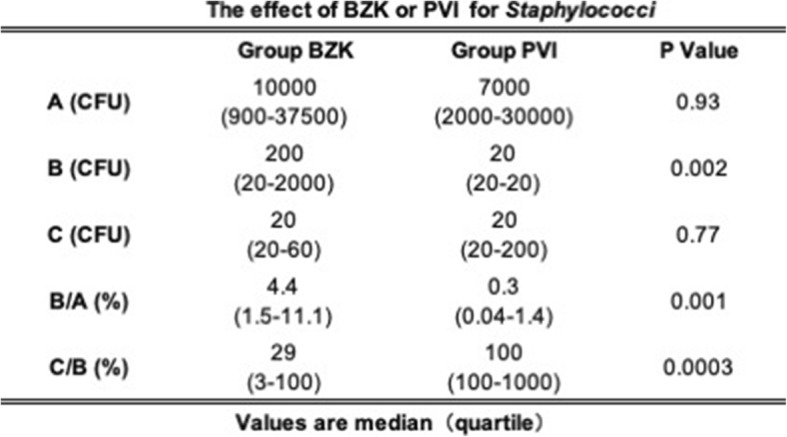
The effect of BZK or PVI for Staphylococci

Tables 4 and 5 show patient distribution regarding the change in the number of bacteria after disinfection. Compared to BZK, more patients with PVI disinfection displayed increased numbers of bacteria after extubation in both media.

**Table 4:**
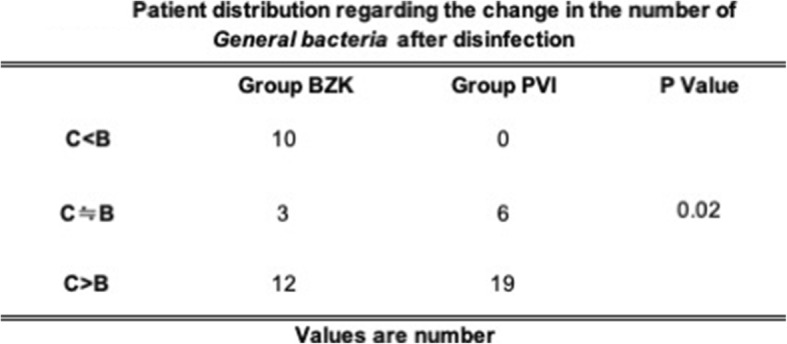
Patient distribution regarding the change in the number of General bacteria after disinfection

**Table 5:**
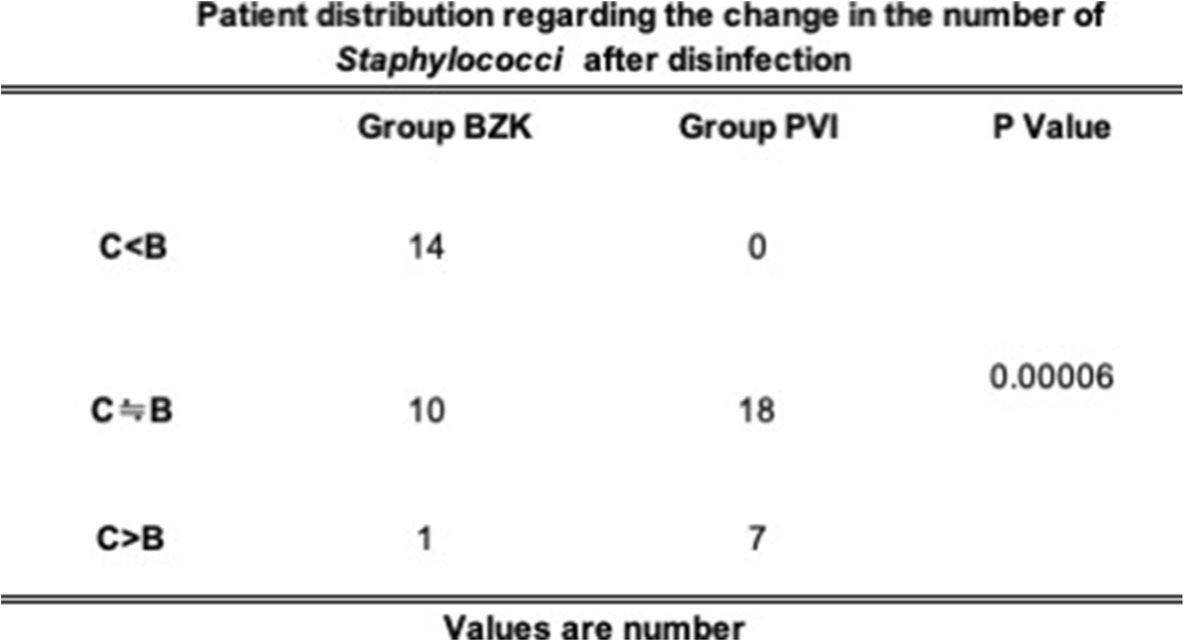
Patien distribution regarding the change in the number of Staphylociccu after disinfection

The MICs of BZK and PVI against *S. aureus* were examined (Table 6). BZK inhibited *S. aureus* when diluted up to 2^9^ times, but PVI only inhibited *S. aureus* when diluted up to 2^6^ times.

**Table 6:**
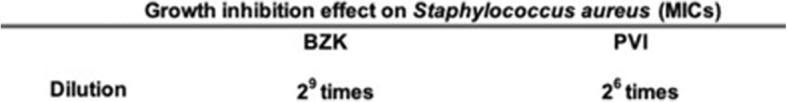
Growth inhibition effect of *Staphylococcus aureus* (MICs)

## Discussion

Previous reports have discussed the development of bacteremia due to bacterial flora associated with the upper respiratory tract (hereafter referred to as “URT”) during treatment [3, 14–17]. Notably, during tracheal intubation, there is a high possibility for the bacterial flora in the nasopharyngeal region to gain access to the trachea. During intubation, host defense mechanisms that remove bacterial pathogens from URT, which would subsequently enter the lower respiratory tract, are impaired. In addition, the frequency of bacteremia after nasotracheal intubation is not related to the use of drugs for blood vessel contraction or the degree of trauma during the procedure [11].

Based on the hypothesis that *Staphylococcus* sp. is an important etiological agent associated with NTI, we used MTS, a selective medium for *Staphylococcus* sp., in this study. Although yellow colonies were frequently isolated, some white colonies were also isolated. On Gram staining of selective specimens, because the yellow and white colonies obtained appeared to be gram-positive cocci forming grape-like clusters, they were considered to be *S. aureus* and *S. epidermidis*, respectively.

In this study, the main bacterial isolates obtained from the nasal cavity were *S. aureus* and *S. epidermidis*. Because *Staphylococcus* sp. causes endocarditis [18], disinfection of the nasal cavity prior to nasotracheal intubation is extremely important to reduce the bacterial load.

Following antisepsis of the nasal cavity, the swab sampling the mucous membrane was placed in 40 ml of saline. The swab head can absorb approximately 0.135 ml of fluid. When the same volume of antiseptic (BZK or PVI) is absorbed by a swab during mucosal membrane swabbing and placed in 40 ml of saline, the sample is diluted 296-fold. Although *S. aureus* MICs of BZK and PVI were achieved after 512-fold and 64-fold dilutions, respectively, no antibacterial effect was expected theoretically because PVI was diluted below the MIC. However, the initial disinfection efficacy of PVI was higher than that of BZK, suggesting that dilution does not affect disinfection efficacy.

Because PVI has wide-spectrum disinfection properties with low levels of irritation, it is used for local applications during surgery and for infections of the oral and vaginal mucosa. Although PVI displays a rapid disinfection efficacy at low concentrations (approximately 0.1%) under experimental conditions, it can lose significant disinfection efficacy in the presence of organic matter; therefore, a 5–10% concentration is used in clinical settings. It is reported that 10 min was required for appearance of the bactericidal effect of PVI in clinical application, which organic matter was present [19]. However, we intubated immediately (1 to 2 min) after the disinfections in this study, but B/A values indicate that PVI was superior to BZK for initial disinfection. Our results suggest that reconsideration is necessary for PVI usage including an incubation time after application. Additionally, it does not seem to wait for 10 min after PVI treatment in general application. As mentioned above, PVI normally exhibits sufficient disinfection effect by waiting for 10 min after disinfection.

In this study, although we intubated 1–2 min after disinfection with PVI, the initial disinfection effect was sufficient. However, if intubation was performed 10 min after disinfection, it may have been possible to suppress bacterial growth in C by exerting the original disinfection effect.

BZK continued to demonstrate a high disinfection efficacy following extubation, whereas increased bacterial levels were found after PVI disinfection. Because the clinical samples following extubation represent specimens incide the endtracheal tube, re-contamination due to bacterial flora in the nasal cavity during extubation is considered to be extremely unlikely. Therefore, the increased bacterial levels detected after extubation indicated that the growth of bacteria originally introduced into the trachea at the time of intubation develops even in a short intubation time during surgery and the disinfection effect of PVI is not sustained.

This study clearly showed that the disinfection efficacy of BZK was sustainable. In vitro, BZK inhibited the growth of *S. aureus* even at a dilution of 2^9^, whereas PVI inhibited growth at a dilution of 2^6^. Although both disinfectants were inactivated or diffused/diluted over time, BZK maintained the threshold concentration and displayed antimicrobial effects longer than PVI; therefore, BZK appeared to show a better sustained effect.

There are some limitations of this study. First, with respect to bacterial counts, there were major differences in callosity among patients. There are many reasons for the variation in callosity. The level of mucosal membrane dryness in the nasal cavity can affect callosity. In the future, it may be necessary to adjust for mucosal membrane dryness prior to specimen collection. Second, multiple practitioner were participated in this study. Prior to beginning the study, disinfection methods were standardized as much as possible; however, disinfection efficacy did not account for differences among individuals. Although this study demonstrated that PVI showed immediate effects, the results may have differed if one healthcare person performed all procedures.

Third, since we only disinfect the nasal cavities and inferior nasal passages, we cannot rule out that naso- and oro-pharyngeal contamination during intubation contributed to our results. We also have not performed blood tests before and after disinfection. Therefore, we may have had to discuss more solid evidence based on blood test for bacteremia.

Forth, since our research is a single-institutional research, longitudinal, multicentric, large population randomized controlled studies comparing the disinfection effects of variety of disinfectants over variety of microorganisms may be necessary to derive a valid conclusion.

## Conclusion

We investigated the disinfection effects of benzalkonium chloride and povidone iodine when used for disinfection in nasotracheal intubation. Although both disinfectants were inactivated or diffused/diluted over time, benzalkonium chloride maintained the threshold concentration and displayed antimicrobial effects longer than povidone iodine. Benzalkonium chloride can be used for creating a hygienic nasotracheal intubation environment with sustained sterilizing effects.

## Data Availability

The datasets analysed during the current study are available from the corresponding author on reasonable request.

## Abbreviation

ASA-PS: American Society of Anesthesiologists-physical status
BHI: Brain heart infusion agar
BZK: Benzalkonium chloride
CFU: Colony-forming units
MICs: Minimum inhibitory concentration
MTS: Mannitol Salt Agar
NTI: Nasotracheal intubation
PVI: Povidone iodine
URT: Upper respiratory tract
